# Risky monetary behavior in chronic back pain is associated with altered modular connectivity of the nucleus accumbens

**DOI:** 10.1186/1756-0500-7-739

**Published:** 2014-10-20

**Authors:** Sara E Berger, Alexis T Baria, Marwan N Baliki, Ali Mansour, Kristi M Herrmann, Souraya Torbey, Lejian Huang, Elle L Parks, Thomas J Schnizter, A Vania Apkarian

**Affiliations:** Department of Physiology, Feinberg School of Medicine, Northwestern University, 300 E. Superior St, 60611 Chicago, IL USA; Department of Rheumatology, Feinberg School of Medicine, Northwestern University, 300 E. Superior St, 60611 Chicago, IL USA; Departments of Anesthesia and Surgery, Feinberg School of Medicine, Northwestern University, 300 E. Superior St, 60611 Chicago, IL USA

**Keywords:** Nucleus accumbens, Connectivity, Resting state, Monetary risk, Chronic back pain

## Abstract

**Background:**

The nucleus accumbens (NAc) has a well established role in reward processing. Yet, there is growing evidence showing that NAc function, and its connections to other parts of the brain, is also critically involved in the emergence of chronic back pain (CBP). Pain patients are known to perform abnormally in reward-related tasks, which suggests an intriguing link between pain, NAc connectivity, and reward behavior. In the present study, we compared performance on a gambling task (indicating willingness to risk losing money) between healthy pain-free controls (CON) and individuals with CBP. We then measured modular connectivity of each participants’ NAc with resting state functional MRI to investigate how connectivity accounts for reward behavior in the presence and absence of pain.

**Results:**

We found gain sensitivity was significantly higher in CBP patients. These scores were significantly correlated to connectivity within the NAc module defined by CON subjects ( which had strong connections to the frontal cortex), but not within that defined by CBP patients ( which was more strongly connected to subcortical areas). An important part of our study was based on the precedence that a range of behaviors, from simple to complex, can be predicted from brain activity during rest. Thus, to corroborate our results we compared them closely to an independent study correlating the same connectivity metric to impulsive behaviors in healthy participants. We found that our CBP patients were highly similarin connectivity to this study’s highly-impulsive healthy subjects, strengthening the notion that there is an important link between the brain systems that support chronic pain and reward processing.

**Conclusions:**

Our results support previous findings that chronic back pain is accompanied by altered connectivity of the NAc. This lends itself to riskier behavior in these patients, a finding which establishes a potential cognitive consequence or co-morbidity of long-term pain and provides a behavioral link to growing research showing that chronic pain is related to abnormal changes in the dopaminergic system.

## Background

The mesolimbic system, and the NAc in particular, has traditionally been viewed as the brain’s primary reward circuitry, and is commonly framed in terms of its functions regarding motivation, reward-valuation, and pleasure-seeking. However, a growing body of literature now suggests that this system has roles which expand beyond monovalent hedonic processing to also encompass aversive learning, including in the contexts of fear, anxiety, and pain [1–3]. Recent research has shown various changes in the reward circuitry, specifically in connections to/from the NAc of individuals with chronic back pain (CBP) [4, 5]. CBP patients exhibit NAc activity that is distinct from healthy subjects with the offset of an acute painful stimulus [6]. Additionally, we and others have identified altered structural and functional abnormalities within this circuitry in these patients, in particular in the connectivity between NAc and prefrontal regions [5, 7, 8]. The medial prefrontal cortex (MPFC) is known to have increased activity in CBP patients [9], and the synchronous activity between this area and the accumbens is highly predictive of transition from a subacute to a chronic pain state one year later. Additionally, people with a variety of chronic pain conditions are known to perform abnormally in tasks designed to engage reward-valuation, motivation, and decision-making circuitry [10–12]. It is unclear, however, how pain-related alterations in these systems factor into reward-behavior. Here, we provide insight into this question by examining the link between reward-oriented decision making, functional brain connectivity, and CBP.

To study decision-making in the context of reward, we use a well-established loss aversion monetary gambling task. Loss aversion is the phenomenon described in prospect theory by which losses have the tendency to have a greater hedonic impact than comparable gains in mixed gambles [13–16]. On average, healthy individuals are roughly two times as subjectively sensitive to losses as they are to gains, such that they would need a potential gain of at least $100 to make up for a potential loss of $50 [14]. This aversion has been robustly demonstrated for a variety of factors – including money and amounts of objects – and has also been found to be stable within subjects across both risky and riskless contexts. [13]. Additionally, being less loss averse – that is, having lower sensitivity to losses and/or increased sensitivity to gains - has been associated with a variety of behavioral and neuropsychiatric disorders , as well as with a general increased probability for risk-seeking and impulsive actions [13, 14, 17, 18]. Thus, performance on this task is thought to be indicative of a person’s likelihood to take risks.

To investigate the relationship between risk behavior and neural activity, we then compare functional brain activity between groups during the gambling task. As a growing body of evidence supports the notion that resting brain activity (which is a measure of synchronous activity between different brain regions during a task-free state) predicts a variety of simple and complex behaviors, we also correlate our subjects’ behavior during this task to their resting state brain connectivity. Because the task scan results showed no differences in activity despite the behavioral differences (refer to Results section), we concentrate primarily on group disimilarities in resting state connectivity. The advantages to measuring resting as opposed to task-based activity is that (1) it is robust and has been shown to be reproducible within and between subjects [19, 20] and (2) it may be viewed as a *baseline* – that is, the brain’s intrinsic functional repertoire in the absence of an external task or event which reflects a person’s accumulation of experiences and learned behaviors [10, 19, 21–23]. We are interested in the extent to which this baseline might indicate one’s monetary risk-taking tendencies and since chronic pain is associated with a pervasive alteration in resting brain activity [24–26], we suspect that these differences in “baseline” activity will correspond to chronic pain patients’ behaviors. Given that primary targets of functional and structural reorganization in CBP lie within reward-processing circuitry (in particular the NAc), and aberrant decision-making in the context of reward has already been demonstrated in chronic pain conditions, we hypothesize that CBP patients will perform differently (i.e., make riskier decisions) in the loss-aversion task than their healthy counterparts. Moreover, since resting activity has been shown to be not only sculpted by individual experience [23, 27] but also reflective and even predictive of an individual’s decisions in various contexts (including risk and reward) [20, 22, 28], differences in task behavior should correlate to differences seen in the resting state functional connectivity of the reward circuitry in our participants.

To corroborate our results, we closely compared them to an independent study using an identical brain connectivity metric that predicted impulsive tendencies in healthy subjects (which we refer to as the “Davis study” [29]). In this study, resting state modular connectivity (which isthe functional grouping of many brain regions based on their synchronicity) was used to measure how different areas of the brain interact with each other over the length of a scan. Although grouped in the same family of functional connectivity metrics such as independent component analysis, seed-based connectivity, and a wealth of graph-theoretic measures, modular connectivity used here was advantageous in that it allowed for many brain regions to be grouped together as a single network without setting arbitrary correlation thresholds. The Davis study found modular connectivity was highly correlated with impulsivity, with the most impulsive individuals exhibiting relatively less connectivity between the NAc and midline prefrontal regions [29]. As NAc-frontal cortex connectivity is of particular importance in CBP, we were interested in how the NAc modules of our CBP patients compared to those of healthy individuals across the impulsivity spectrum.

## Results

**Figure 1:**
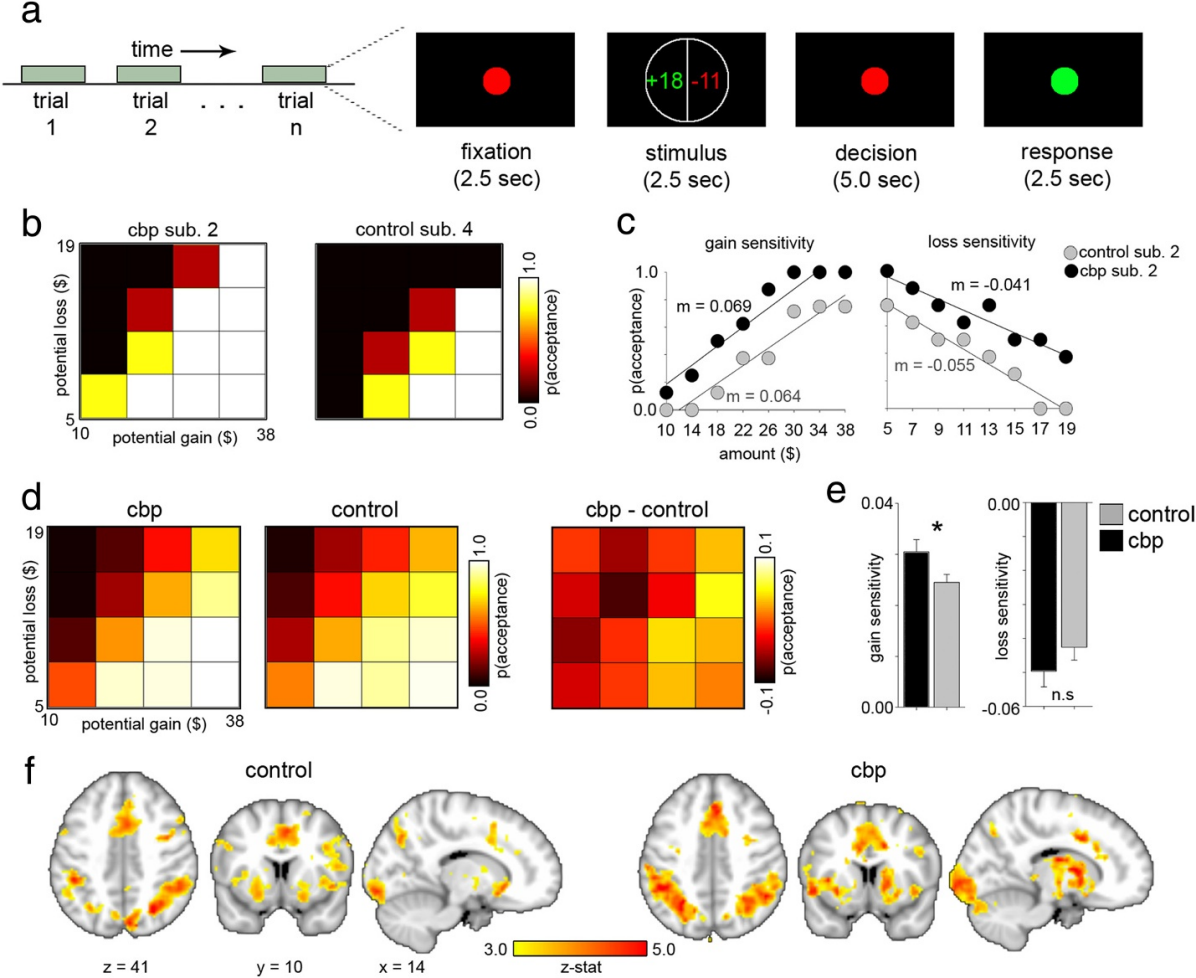
**Monetary decision making is more impulsive in CBP relative to CON, seen in behavior but not in task brain activity. a)** A depiction of the task is given. When the stimulus offer was shown, participants decided whether the possibility of winning the amount in green was worth possibly losing the amount in red. Each trial lasted 12.5 seconds, with an inter-trial interval of 10 to 12.5 seconds. **b)** Loss-aversion matrices are shown for 2 subjects. Notice the CBP patient was more likely to accept a monetary offer than the CON subject, with complete acceptance at the highest potential gain. **c)** Gain and loss sensitivity curves are shown for the same 2 subjects. Each point represents the probability of accepting an offer withr each potential gain or loss. The number “m” is the slope of the fitted line, indicating their gain or loss sensitivity. **d)** Mean loss aversion matrices for each group are shown. The right-most matrix is the difference between the group means, highlighting the higher probability of acceptance for higher gains in CBP. The color bar expresses a different range of probabilities, where negative values indicate a higher probability of acceptance in CON, and positive values indicate higher probability for CBP. **e)** The bar plots show each group’s mean gain and loss sensitivity scores. The error bar represents the standard error. Only gain sensitivity is significantly different (p < 0.05), with CBPs exhibiting a higher likelihood of accepting monetary offers when larger potential gains are involved. **f)** Regions with significant activation for task versus baseline for CON and CBP are shown, with Z-statistic thresholded at 2.3 (shown here >3.0) and results whole-brain cluster corrected at p < 0.05. The statistical contrast map between activations for CONs and CBPs was empty, even before correction for multiple comparisons.

Overall, CBP patients were more likely to accept offers with higher potential gains during the loss-aversion task, which is visually explained in Figure 1a-c. This can be seen in the group mean decision matrices in Figure 1d. Of the three scores compared, only gain sensitivity was significantly different between CBP and CON groups (p < 0.05, Figure 1e). CBPs (3.11 × 10^−2^ ± 6.11 × 10^−3^) had higher gain scores than CONs (2.53 × 10^−2^ ± 6.79 × 10^−3^). The group difference remained stable (p < 0.05) for the smaller subset of participants who had the entire set of imaging data (CBPs: 3.05 × 10^−2^ ± 7.18 × 10^−3^ and CONs: 2.45 × 10^−2^ ± 6.47 × 10^−3^). The result indicates a greater likelihood that CBP patients would accept offers with larger potential monetary gains *even if* they could potentially lose larger amounts of money. This difference in behavior could not be attributed to pain during performance, as there was no correlation between gain scores and pain ratings on the day of the task (r = 0.058, p = 0.882). Average reaction times during the task were also not significantly different between groups (CBPs: 479.12 ± 93.3 ms and CONs: 485.24 ± 103.8 ms; p = 0.88).

### Connectivity

Our initial imaging analysis was focused on brain activity related to the task, using a general linear model to generate a group contrast between CBP and CON participants. After creating activation maps showing significant increases in activity during the task versus during inter-trial intervals for each group (Figure 1f), we compared the two group maps. Nothing survived correction for multiple comparisons, nor were there significant differences in activity before this correction – the contrast map was empty in both instances.

**Figure 2:**
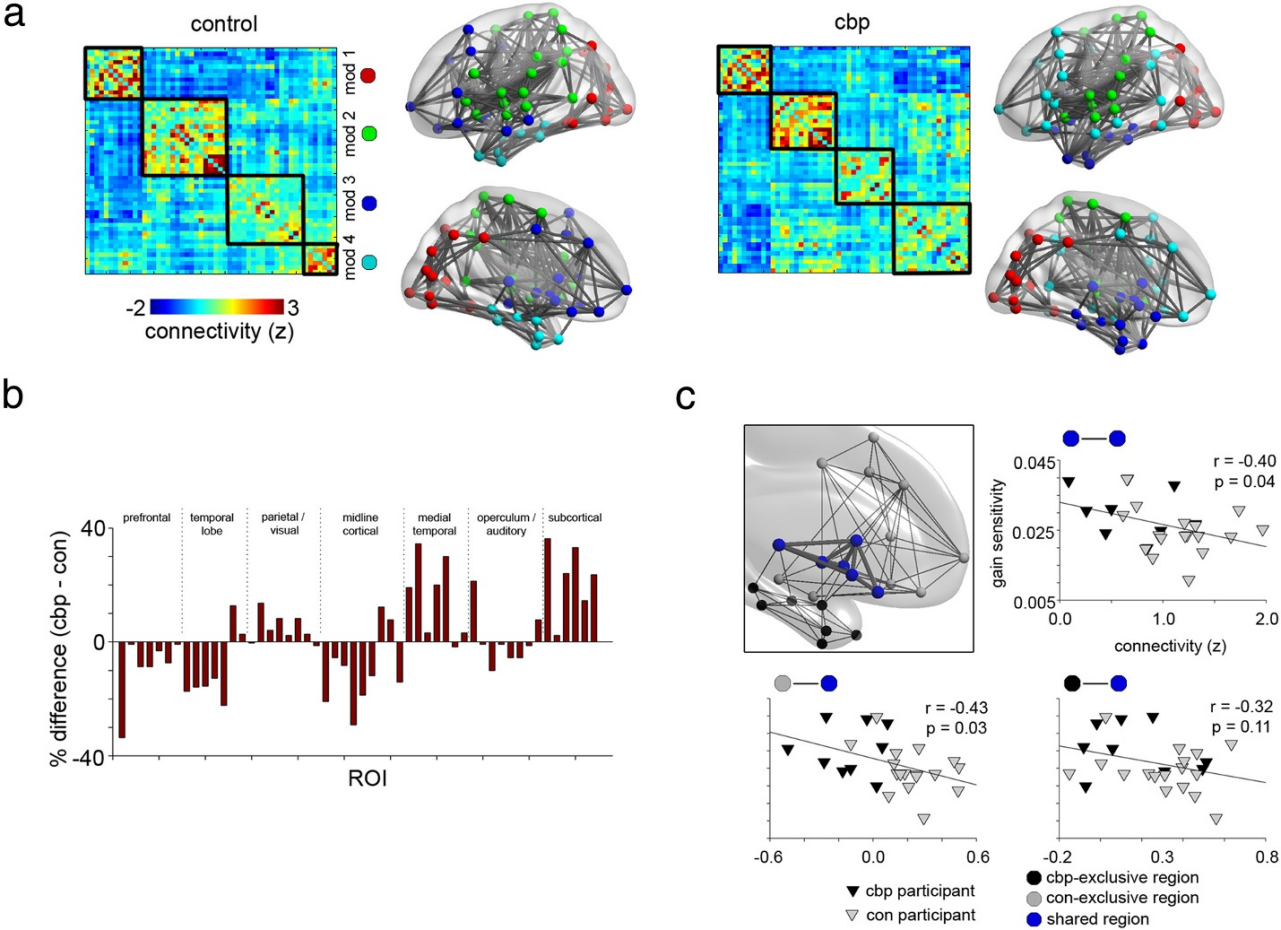
**NAc modular connectivity includes more subcortical regions in CBP and more frontal regions in controls. a)** The biggest difference in modular structure of the group mean connectivity matrices lies between modules 3 (the NAc module) and 4. Connectivity matrices are labeled as they best align to those previously defined [29]. Shown are the lateral and medial views of the brain. For CON, the NAc module integrates more frontal regions, while module 4 is primarily subcortical. In contrast, for CBP, the NAc module integrates subcortical structures, while moduIe 4 includes mostly frontal and parietal regions. **b)** Each brain region (see Table 1) is assigned its likelihood of membership with the NAc module based on the percentage of participants including that region with this module. The bar plot shows the difference between the groups. A greater percentage of CBPs integrated subcortical and medial temporal regions with the NAc, while more controls integrated midline cortical, temporal lobe, and prefrontal regions. **c)** The NAc module is divided into shared and exclusive regions. Mean connectivity across these regions are correlated to gain sensitivity scores. The inset plot indicates regions that both CON and CBP integrate into the NAc module (blue nodes), based on each groups’ mean connectivity matrix. Gray nodes indicate those regions that are exclusively integrated to the CON’s NAc module, while black nodes are exclusive to CBP. Scatter plots illustrate the Pearson correlation between gain sensitivity scores and the mean connectivity between all nodes within each group of regions. The only plot that is not significantly correlated is with the blue to black nodes, suggesting the fully integrated NAc module in CBP cannot account for gain sensitivity, whereas that of controls can. Black lines in all maps indicate functional links between regions thresholded at a whole-brain link density of 0.2.

We therefore turned our focus to resting state connectivity. Note, from here on out, we use the terms “connectivity” and “modular connectivity” interchangeably. Our goal was to determine how modular connectivity of the NAc was related to impulsive monetary behavior, and whether this relationship was altered in CBP. We designate the nomenclature of modules according to a previous study examining modular connectivity and impulsive behavior [29]. In that study, for their “intermediate” impulsive participants, “module 1” consisted primarily of visual processing regions; “module 2” was mostly somatosensory, motor, and auditory; “module 3” (also labeled “subcortical drive 1”) was the NAc module, but also contained frontal and temporal lobe regions; and “module 4” (also labeled “subcortical drive 2”) consisted mostly of medial temporal lobe structures including the amygdala and hippocampus. As modularity algorithms assign numbers to modules arbitrarily, we re-labeled our modules to best fit this structure. Further, for the remainder of the paper, we refer to module 3 as the “NAc module”, as it is our main focus. Figure 2a illustrates the modularity of the mean connectivity matrix for each group, showing in general that the CON group followed the whole-brain modular structure mentioned above, while the CBP deviated in the NAc module and module 4. The CBP NAc module integrated more subcortical regions, including the hippocampus and amygdala, but it was segregated from the frontal regions included in the CON group. Module 4 was also quite different from that of the CON group, consisting mainly of frontal and parietal regions with essentially no medial temporal regions. Table 1 provides the modular designation of each brain region for both groups. The main finding here is that the CBP NAc module was more integrated with other subcortical structures, and less with frontal regions – the opposite of the CON group. The total number of modules in the brain was not significantly different between groups (CBP = 3.68 ± 0.57, CON = 3.55 ± 0.59).

**Table 1:** **List of ordered brain regions in Figure** 2 **b**

	Region	CBP	CON
Prefrontal	Frontal_Pole*	4	3
	Insular_Cortex	2	2
	Superior_Frontal_Gyrus	4	3
	Middle_Frontal_Gyrus	4	3
	Inferior_Frontal_Gyrus_pars_triangularis	4	3
	Inferior_Frontal_Gyrus_pars_opercularis	4	2
	Precentral_Gyrus	2	2
Temporal lobe	Temporal_Pole	3	4
	Superior_Temporal_Gyrus_anterior_division	2	2
	Superior_Temporal_Gyrus_posterior_division	2	2
	Middle_Temporal_Gyrus_anterior_division	4	3
	Middle_Temporal_Gyrus_posterior_division*	4	3
	Middle_Temporal_Gyrus_temporooccipital_part	4	2
Parietal/visual	Inferior_Temporal_Gyrus_temporooccipital_part	4	1
	Postcentral_Gyrus	2	2
	Superior_Parietal_Lobule*	2	2
	Supramarginal_Gyrus_anterior_division	2	2
	Supramarginal_Gyrus_posterior_division	4	2
	Angular_Gyrus	4	2
	Lateral_Occipital_Cortex_superoir_division	1	1
	Lateral_Occipital_Cortex_inferior_division	1	1
	Intracalcarine_Cortex	1	1
Midline cortical	Frontal_Medial_Cortex	4	3
	Juxtapositionalobule_Cortex	2	2
	Subcallosal_Cortex	3	3
	Paracingulate_Gyrus*	4	3
	Cingulate_Gyrus_anterior_division	4	3
	Cingulate_Gyrus_posterior_division	1	1
	Precuneous_Cortex	1	1
	Cuneal_Cortex	1	1
	Frontal_Orbital_Cortex	4	3
Medial temporal	Parahippocampal_Gyrus_anterior_division	3	4
	Parahippocampal_Gyrus_posterior_division	3	4
	Lingual_Gyrus	1	1
	Temporal_Fusiform_Cortex_anterior_division*	3	4
	Temporal_Fusiform_Cortex_posterior_division*	3	4
	Temporal_Occipital_Fusiform_Cortex	1	1
	Occipital_Fusiform_Gyrus	1	1
	Frontal_Operculum_Cortex*	4	2
Operculum/auditory	Central_Opercular_Cortex	2	2
	Parietal_Operculum_Cortex	2	2
	Planum_Polare	2	2
	Heschls_Gyrus_(includes_H1_and_H2)	2	2
	Planum_Temporale	2	2
	Supracalcarine_Cortex	1	1
	Occipital_Pole	1	1
Subcortical	Thalamus*	3	3
	Caudate	3	3
	Putamen	3	3
	Pallidum	3	3
	Hippocampus*	3	4
	Amygdala	3	4
	Accumbens	3	3

Numbers on the right indicate to which module each region belonged based of the average connectivity matrix for each group, and correspond to those shown in Figure 2a. Notice, modules 1 and 2 are mostly matched across regions. Modules 3 (NAc module) and 4 are the most different. Regions are listed in the order they appear in the Harvard-Oxford atlas, and divisions placed between them are simply to illustrate under which category most of them fit. Bolded regions with asterisks indicate areas that are significantly different (p < 0.05) between CBPs and CONs in their % membership with module 3 (NAc module) as shown in Figure 2b.

Figure 2b illustrates the percentage of participants that integrate each brain region into the NAc module. The CON group has roughly 15% – 20% more participants than CBP that include prefrontal, temporal lobe, and midline cortical structures into the NAc module. Integration of medial temporal and subcortical structures into the NAc module, on the other hand, was 20% to 30% higher in CBP. A visual depiction of this is shown in the inset of Figure 2c, where regions shown in blue are common members of the NAc module in both groups – determined by each group’s average connectivity matrix. Those shown in gray and black are exclusive to either CON or CBP, respectively. Further, here we see that the mean connectivity across blue regions, and between blue and gray regions, is significantly negatively correlated to gain sensitivity (p < 0.05). This is not the case with connectivity between blue and black regions (p = 0.11). In other words, all our participants’ gain sensitivity scores can be accounted for by NAc connectivity, if we define that connectivity using only our healthy subjects. This is not the case if we define NAC connectivity with only our CBP patients. This is a key finding in our study, and suggests that the connectivity the CBP group lacks (to the frontal areas) may influence impulsive behavior.

**Figure 3:**
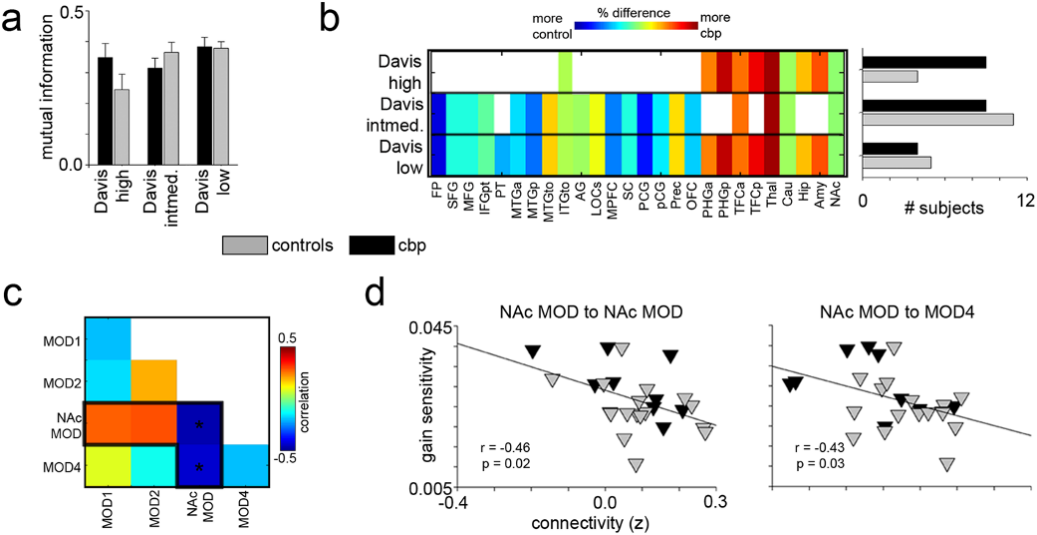
**Brain modular connectivity in CBP matches that observed in high impulsivity. a)** Mutual information between whole-brain modular structure of each participant and those previously described (“Davis templates”, [29]) for healthy individuals with high, intermediate, and low-impulsive behavior. Although mutual information between CON and CBP participants was not significantly different, CBP expressed a more similar modular structure to highly impulsive individuals. Bars indicate group mean, error bars are standard error. **b)** The percentage of participants that integrated each region with their NAc module (established from the union of NAc modules for all Davis templates) is shown. Colors toward the red end of the spectrum indicate a greater percentage of CBPs included a specific region in their NAc module, whereas those toward the blue end indicate a greater percentage of CONs; white indicates that a region was not part of that specific template. For regions that were part of the NAc module in the high impulsivity template, there was a greater percentage of CBPs with the same regions in their NAc modules, whereas a higher percentage of CONs integrated the intermediate regions into their NAc. The bar plot shows the number of subjects whose NAc module overlaps the most with each Davis NAc module, illustrating the same results as the color plot. **c)** Using modules delineated by the intermediate-impulsivity template, the correlation matrix indicates the Pearson correlation of gain sensitivity to the mean connectivity across modules. The most extreme correlations, outlined in black, are those that take the NAc module connectivity into account. Asterisks are significant (p < 0.05), and correspond to the plots shown in D. **d)** Scatter plots of the significant correlations from C are shown. Only gain sensitivity and connectivity of the NAc module to itself and to module 4 were significantly correlated (p < 0.05).

Given the findings so far, we questioned whether our CBP participants’ brains were similar to healthy individuals with impulsive tendencies. As our healthy controls exhibited lower monetary risk in general, we tested the similaritybetween our participants and those of an independent study (the Davis study) examining modular connectivity and impulsivity in a healthy population [29]. Using the modules from subjects deemed “low”, “intermediate”, or “high” impulsivity in [29] as templates (from here on out referred to as *Davis templates*), we measured the mutual information between these templates and the modular structures from each of our participants. Mutual information ranges from 0 to 1, with a “1” meaning that modular structure between 2 brains is identical. ANOVA on ranks determined mutual information was not different between CON and CBP groups. However, mutual information with the high-impulsivity Davis template was most different between groups, with CBP exhibiting 0.35 ± 0.21 and CON exhibiting 0.25 ± 0.22 mutual information. Additionally, our CON participants showed the least similarity to the high-impulsivity template. Although not significant, the mean difference indicates CBP patients tend to have general modular connectivity that is more similar to highly impulsive individuals compared to our CON participants (Figure 3a).

When we examined the NAc module alone, we found that CBPs were more similar to the highly impulsive Davis participants. CONs, on the other hand, were more similar to the intermediately impulsive individuals. Figure 3b depicts this in 2 ways. First, similar to Figure 2b, we determined the percentage of participants that integrated each brain region into their own the NAc module. The color plot is a narrowed-down version of Figure 2b, including only brain regions that were within the NAc modules of all Davis templates (all regions spanning the NAc modules of low, intermediate, and high templates). One can see the NAc modules of CBP patients were more similar to those of the highly impulsive Davis participants. On the other hand, our CON participants had NAc modules that were more similar to those of the intermediate- and low-impulsivity Davis participants. The bar plot to the right illustrates the number of participants in each group that had the greatest overlap with the Davis NAc modules. The distribution of the bar plot shows that CBP participants tended to have NAc modules more similar to high impulsivity individuals, followed by intermediate, then low. On the other hand, CONs were most similar to the intermediate, followed by low, then high. Overall, this result suggests that the connectivity of the NAc in CBPs is very similar to that of highly impulsive individuals, whereas our CONs have similar connectivity to individuals with intermediate impulsivity.

The Davis study highlights that impulsivity was correlated to connectivity across modules, rather than within modules. We wanted to test this in a similar fashion by correlating our gain sensitivity scores to cross-module connectivity. For the sake of consistency with the Davis study, we defined our modules identically to theirs. Because most of our participants modules were most similar to those of the intermediate Davis participants, we concentrated on that template. The correlation matrix is shown in Figure 3c, where correlations with the NAc module are outlined in black. Only the connectivities of the NAc module to itself and to module 4 were significantly correlated to gain sensitivity (p < 0.05). Figure 3d demonstrates a negative relationship, suggesting that the more tightly the nodes of the modules are integrated (which includes prefrontal, NAc, and amygdala), the less gain sensitive the individual. Overall, we show that 1) the modular connectivity of the brain in our CBP patients is similar to highly impulsive individuals, whereas our CON participants are more similar to intermediately impulsive individuals, 2) the connectivity within and across modules (specifically with those involving the NAc) correlates to reward behavior, and 3) these correlations are stable whether modular structure is determined by our data or a completely independent data set. For all connectivity analyses, regressing out age and sex did not change our results.

## Discussion

Here we show that monetary reward behavior in CBP patients differs from healthy individuals. Whereas both groups demonstrate equal sensitivity to monetary loss and thus have an equal sensitivity to aversive consequences, CBP patients are significantly more sensitive to monetary gains, and in turn are likely to take greater risks when the opportunity for receiving larger sums of money presents itself or when a big enough reward is offered. We show further that the resting brains of these two groups differ in their modular connectivity, notably in regions that are integrated with the NAc. Healthy participants tended to have more connectivity between the NAc and frontal regions, while the NAc of CBP patients was more connected to subcortical structures. Moreover, when we compared the modular connectivity of our participants’ brains to those of another study with a range of impulsive subjects, we found that CBP patients resembled those highly-impulsive subjects, while our CON participants were more similar to intermediately-impulsive subjects. Finally, we showed connectivity between the NAc and frontal regions was more correlated to reward behavior than connectivity between the NAc and subcortical structures. We thus propose that the lower gain sensitivity of our healthy participants is likely due to functional NAc-frontal connections that are lacking in CBP patients.

These results fit well with current literature regarding risk, reward evaluation, and decision making. The steps leading up to a decision may involve minimizing and negotiating consequences, problem solving, or controlling one’s emotions in order to think more clearly. All of these processes involve top-down modulation of subcortical reward and emotion centers by the brain’s reflective and executive systems including the frontal, anterior cingulate, and insular cortices [28, 30, 31]. Simultaneously, subcortical areas (e.g., the NAc, amygdala, and hippocampus) can also exert bottom-up influences on this process, generating strong positive or negative emotional states which also act to bias or steer the decision [28]. Many neuropsychological and behavioral disorders that involve a lesion between frontal and subcortical circuitry or an impairment in the functioning of these regions (e.g., Attention Deficit Hyperactivity Disorder (ADHD), pathological gambling, trichotillomania (TTM), specific focal brain injuries, and drug addiction) often present with symptoms that encompass a greater difficulty in making decisions and include a lack in premeditative skills and a decreased suppression of automatic thoughts [30, 32–36]. These differences and/or changes in decision-making often lead to risk-taking and impulsivity, behaviors and traits that these conditions are known for. For example, individuals with ventromedial PFC (vmPFC) damage often exhibit altered decision-making processes in the presence of financial risk, such that they become more attentive to rewards and gains and less attentive to potential losses, [30, 33]. People with various kinds of substance abuse disorders have also been shown to act more impulsively, performing very similarly to vmPFC patients in a variety of gambling tasks [37], and functional neuroimaging has shown that substance abuse is also associated with changes in activity in and between frontal and subcortical limbic areas [30, 37]. Importantly, changes in resting state functional connectivity within the mesolimbic circuitry have also been shown to be anticorrelated to impulsivity [29, 38], and activation of the mPFC during risky decisions has been shown to be negatively correlated to a healthy person’s individual risk preference [39, 40]. All of these findings can help explain why we see a negative correlation between NAc-frontal connectivity and gain sensitivity in our study – what they suggest is that more connections from frontal regions to the NAc allow for more inhibition of riskier tendencies and in turn make the person less sensitive to monetary gains.

Loss aversion is a complicated phenomenon involving the interplay of many brain systems. Thus, while we focus primarily on the relationship between prefrontal regions and the ventral striatum in loss aversion, the roles of other areas like the amygdala, insula, and hippocampus (all of which help to detect and plan for risks) should not be ignored. The amygdala in particular has a role in aberrant risk-taking and loss perception. Many forms of impulsivity (monetary and otherwise) in drug addicts [30, 38, 41], pathological gamblers [42], people with TTM [32], and healthy subjects [43] have been linked to amygdala activity, volume [44], and/or connectivity from amygdala to striatum or frontal regions[45]. Amygdala differences have also been directly tied to monetary risk – in one study, subjects who had structural damage to the amygdala, experienced an elimination of behavioral loss-aversion and increased their selection of monetary gains [46]. These findings may help explain why our CBP patients’ NAc module was more integrated to subcortical regions including the amygdala, than was that of our CONs. Our results provide solid evidence that amygdala connectivity is altered in CBPs, and specifically point to a relationship between gain sensitivity and its connections to the NAc. However, the details of its connections to the whole brain and how this corresponds to risk-taking is not fully covered in this study. A focused examination relating amygdala function/structure to impulsivity and chronic pain would be of high interest.

The extent to which our results suggest an inability to process monetary risk *specifically,* and in turn reflect a dysfunctional reward system in CBP, needs discussion. This is because reward perception and action may also be altered by an overall lack of or difference in emotional awareness during decisions [47] or by changes in attentional mechanisms [48, 49]. Importantly, both of these alternatives might be due to our participants either having a history of chronic pain, which would affect the brain’s circuitry, and/or having a presence of pain while they are performing the task, which might change their scores. Regarding the first possibility, changes in functional connectivity between the regions discussed here have also been implicated in disorders characterized by intense apathy, defined as an “absence or lack of feeling, emotion, interest, or concern”, a “lack of motivation”, and in turn, a “reduction of voluntary, goal-directed behaviors” [50]. For example, individuals with these clinical conditions (which included athymormia and a subset of Parkinson’s patients) who score highly on measurements of apathy have been shown to have disruptions in the connectivity between either orbital-medial PFC and basal ganglia or between their dorsolateral PFC and basal ganglia, depending upon how their apathy affects their daily lives [50]. It could be that for CBP patients, being in constant pain for many years has changed how they process emotions in general (as opposed to those dealing specifically with reward), and this in turn has influenced their response to decisions and contexts involving risk or high emotions. These studies suggest disconnections between frontal and striatal areas dull responses to monetary scenarios (that is, no preference is shown to either avoid losses or seek gains); this, however, is not the case in our CBP patients, who clearly preferred higher potential monetary gains no matter the corresponding monetary loss amounts. Thus, while there certainly seems to be a relationship between reduced connectivity and blunted emotional awareness, it is unclear as to whether the results presented here support this finding.

This leads us to the second possibility mentioned above – that changes in attentional mechanisms or resources might be contributing to changes in connectivity and differences in behavior. Due to the overlap in the network regions involved in both chronic pain and reward, it is possible that the cognitive demand of the ongoing background pain may alter CBP patients’ ability to attend to the task (in this case, to attend to both losses and gains equally) [48, 49], and in turn this may be reflected in our neuroimaging findings. Previous results have shown that CBP patients do *not* appear to have deficits in other types of tasks, including those measuring attention and short-term memory [10]. However, the intensity of the chronic pain experienced by these patients has been shown to correlate negatively with their performance in a different monetary gambling task (Iowa Gambling Task), such that the more pain they were in on the day of the task, the lower their score was (and the riskier they behaved) [10]. Importantly, this relationship between pain intensity and task was not present in our current behavioral results – there was no correlation between gain scores and pain ratings on the day of the task. What these previous findings suggest is that our current behavioral results point to a more specific cognitive deficit related to reward valuation (as opposed to a more general cognitive impairment related to pain’s effect on attention or emotion). More specifically, our results put forward the idea that CBP may not be a deficit of loss aversion per se, as patients did not demonstrate a reduced sensitivity to aversive consequences; rather, CBP’s effect on the reward system may impact gain-seeking specifically, in that patients displayed an increased sensitivity to rewarding consequences, which could also lead to increased risky or impulsive behavior.

Because of its stability across scanning sessions, resting state scans are commonly viewed as measure of ‘baseline’ brain activity, and thus in some respect are analagous to fingerprints or genetic profiles of an individual. Our choice to correlate resting activity to behavior grew out of the idea that decision-making is a stable characteristic of personality, is a constant process that pervades daily life, and it requires many brain systems. It should thus be reflected in baseline activity. This type of analysis is not uncommon, and it has been shown that resting brain activity correlates to behavior and performance in simple cognitive and perceptual tasks [29, 51–55]. Further testament to this idea might also be seen in the growing number of studies indicating altered resting state activity in diseases associated with abnormal behavior such as Alzheimers, Schizophrenia, and chronic pain [25, 56–59]. In the case of our paper, resting state connectivity also provided us with an alternative method to link brain activity with behavior, since we were unable to do so with our task-based scan. Some possible reasons why our GLM analysis failed to bring out differences in task activity during the scan could be due to the set up of the task itself. Limitations include that there may not have been enough trials to be able to get a high enough sensitivity for regions of increased activation (since previous studies have used upwards of 256 trials compared to our 64 [14]). Additionally, the accept/reject binary response option may not have been nuanced enough to capture group differences in brain activation, and instead the utilization of a likert scale on top of their response (to indicate how strongly or weakly they accepted or rejected the offer) would have brought out more subtle differences in neural activity [14].

## Conclusions

Here we show that monetary decision making in CBP patients is more impulsive than healthy individuals. We demonstrate this behavior is reflected in their baseline brain connectivity, with CBP patients lacking connections between the NAc and frontal regions, but exhibiting stronger connections to subcortical structures. Our results are in alignment with a completely independent study, and we show that CBP brains are similar to those belonging to highly-impulsive individuals. We propose that the higher amount of gain sensitivity present in our CBP patients, a finding indicative of an increased likelihood of both risk-taking and impulsivity, is likely due to a lack of NAc-frontal functional connections that are present in healthy participants. This finding is important because it establishes a behavioral link to growing research showing that chronic pain is related to abnormal functional and structural properties – including connectivity - in the dopaminergic system.

## Methods

### Subjects

Twenty-one CON participants (8 females, 36.6 ± 6.94 y.o.) and 22 CBP patients (10 females, 45.9 + − 7.8 y.o.) enrolled in the neuroimaging study. For various reasons, not everyone could complete the full study; all 21 CONs and 13 CBP patients (6 females, 44.2 ± 7.13 y.o.) performed the loss-aversion task scan and a subset of these individuals also received the resting fMRI scan and a T1 anatomical scan as part of the full neuroimaging study (CON N = 18 (5 females), 35.3 ± 5.6 y.o. and CBP N = 9 (5 females, 46.7 ± 6.0 y.o.). The main behavioral analysis was done on the entire cohort of subjects who had completed the behavioral task scan and data were used to verify the consistency of the results in the smaller cohort who had the full battery of scans; all neuroimaging analyses were done on this subset of participants with both the resting state and task-based scans. CBP patients had pain for > 1 year with no other pain co-morbidities, and CON participants had no history of pain. All subjects provided informed consent to procedures that were approved by the Northwestern University Institutional Review Board.

### Loss-aversion task

In the scanner, participants performed a task, which was adapted from a published report [14], to measure their behavior in making monetary decisions. A visual description of the task can be seen in Figure 1a. In order to best emulate a gambling experience with real monetary penalties (and in turn a real element of risk), we wanted people to feel as if they were actually winning or losing money during the task; thus, participants were given $30 in cash at 1–2 weeks before testing and told that it was theirs to keep but that some of this money may be going toward the future gambling task. This approach is similar to that established in previous studies[14, 60] and attempts to minimize the type of risk-seeking that can happen when people receive “free money” or feel as if they are “playing with the house money”[61]. Stimuli were presented using Presentation software, version 14, via a screen attached to the back of the scanner. Trials began with a red dot (2.5 seconds) followed by a display where a monetary offer was presented to them. The offer showed 2 numbers in 2 colors: green indicated the amount of money they could potentially win (a monetary gain) if they accepted the offer, and red indicated the amount of money they potentially could lose (a monetary loss) if they accepted the offer. Participants were told that they had a 50/50 chance for gaining or losing money on all offers, and they were asked to think about whether they would like to take the chance of winning the amount of money in green with the risk of losing the amount of money in red. After 2.5 seconds, the option disappeared and the red dot re-appeared for 5 seconds (decision-making interval). When this red dot switched to green, participants were asked to press a button indicating their decision to “accept” or “reject” the offer (decision-action interval). Participants had 2.5 seconds to press the button, giving them 10 total seconds from presentation of the offer to make their decision. After the decision period, trials were separated with random inter-trial intervals ranging from 10 to 12.5 seconds. Decisions that were made either before the green dot appeared (too early) or after the 2.5 second button press period (too late) were discarded. Potential gains ranged from $10 to $38 in $4 increments and potential losses ranged from $5 to $19 in $2 increments. These values were chosen based on existing literature suggesting that people are about twice as sensitive to monetary losses as they are to monetary gains [14]. Each offer was presented 8 times in different combinations such that no combination was repeated, resulting in 64 monetary offers total, divided over 2 sets of 32 trials, with all combinations presented in a random (as opposed to continuous) order. To make the consequences of the participants’ decisions as realistic as possible, we randomly chose one of their offers and paid them according to their decision. For example, after the offer was randomly chosen, we flipped a coin -- if it came up heads, the subject was paid the amount of money in green in addition to their participation reimbursement; if it was tails, the amount of money in red was subtracted from their reimbursement. If they had previously rejected the randomly chosen offer, they received or lost no extra money. Thus subjects could win up to an extra $38, or receive up to $19 less for their participation.

Decision matrices were generated by calculating the probability of accepting each offer (i.e., how many times out of the 8 presentations of each offer did the participant choose to “accept”), creating an 8×8 matrix for each subject. Matrices were then down-sampled to a 4×4 matrix by doubling the increments of potential gains and losses and averaging the probability within each matrix element. Thus, each element in the 4×4 matrix is composed of the average probability from the corresponding elements of the original 8×8 matrix. An identical approach was taken in [14]. Example decision matrices are shown in Figure 1b.

To calculate behavioral loss aversion, each participant’s data (probability of accepting an offer as a function of potential gain or loss) was fit with a straight line. The resulting regression coefficients served as individual measures of a person’s loss and gain sensitivity (an example is shown in Figure 1c). Although similar to prospect theory, this calculation makes the assumptions of (1) a linear rather than curvilinear function and (2) identical decision weights for choices involving a 50% chance of either winning or losing money; this approach is identical to those used in previous studies [14, 46]. The overall behavioral loss aversion (*λ, lambda*) score for each participant was calculated as the ratio of the (absolute) loss sensitivity to the gain sensitivity (loss/gain). All fits, coefficients, and behavioral scores were calculated using MATLAB version R2010b (MathWorks). Gain sensitivity, loss sensitivity, and lambda scores were each averaged over groups and compared with an unpaired t-test.

### fMRI acquisition

Whole-brain functional MRI data were acquired with a 3 T Siemens TIM Trio whole-body scanner with echo-planar imaging (EPI) capability, using an 8-channel head coil. The following parameters were used to collect multi-slice T2*-weighted echo-planar resting state images: TR = 2.5 s, echo time TE = 30 ms, flip angle = 90°, slice thickness = 3 mm, in-plane resolution = 3.475 × 3.475 mm^2^, number of slices = 36, number of volumes = 244. Resting scans lasted 10 minutes, during which time participants were asked simply to keep their eyes open. Gambling task functional scans were collected described in a previous report [7]; the only differences from resting state parameters were FOV = 256 mm, in-plane resolution = 86 × 72 mm^2^, number of slices = 40, number of volumes = 281. Each subject underwent two consecutive scans like this (lasting about 12 minutes each).

### Anatomical scans

T1-weighted anatomical MRI image were acquired for each subject to aid in registering brain images to standard MNI 2 mm space. Images were collected with the following parameters: TR = 2.3 s, TE = 4.38 ms, flip angle = 8°, FOV = 256 mm, slice thickness = 1 mm, in-plane resolution = 1.00 × 1.00 mm^2^ and number of axial slices = 160.

### fMRI data preprocessing

Functional MRI data were preprocessed using FSL FEAT (FMRI Expert Analysis Tool). Resting state images were subjected to skull extraction, slice-timing correction, bulk head motion correction, spatial smoothing (Gaussian kernel of full-width-half-maximum 5 mm), and a high-pass (150 sec) temporal filter. Bulk head motion was < 3 mm for all subjects. Images were further corrected for motion, cerebrospinal fluid, and white matter using independent component analysis, performed with MELODIC. These artifacts were identified based on their component spatial maps and time courses, and their time courses were then regressed out of the BOLD signal, voxel-wise. Global mean correction was also performed by linear regression of the average time series of all brain voxels from the BOLD signal at each voxel. Task imaging data were preprocessed similarly to resting data with the only difference being application of a high-pass temporal filter of 50 s. Again, after preprocessing, the same sources of noise mentioned above were removed through linear regression.

### Task-based GLM analysis

Task scans were first registered to the subject’s individual T1 structural image and then into standard MNI space. Whole-brain statistical analysis was performed with FSL’s FMRI Expert Analysis (FEAT) tool using a multi-staged approach before final contrast between groups was made. Statistical modeling was first performed separately for the gambling task runs (split between two scans). Although there are many ways we could have approached the analysis for this task (for example, breaking it down into parameters such as gains versus losses, accepts versus rejects, difficulty of decisions, etc. [14]), we were primarily interested in resting state functional connectivity in relation to decision making, and therefore kept this analysis simple, choosing to study overall task-related activation. After applying a high pass temporal filter (50 sec), the regressor of interest was made by convolving a canonical (gamma) hemodynamic response function with a binary vector (created in Matlab) that represented each trial’s onset and duration (+1) and all intertrial intervals (0). The task positive contrasts were obtained for each person for each of the two scans, and the runs were combined in a fixed-effects model for each participant.

A higher-level analysis was executed that combined all sessions for all participants in a given cohort using FSL’s FLAME (FMRIB Local Analysis of Mixed Effects) tool, treating participants as a random effect. Here, a one-sample t-test was performed at each voxel for the contrast. Z (Gaussianised T) statistic images were thresholded using clusters determined by Z > 2.3 and a whole-brain corrected cluster significance threshold of p <0.05. An unpaired two-sample t-test was constructed with FSL’s General Linear Model (GLM) interface to compare positive activity maps between CBPs and CONs, with each cohort represented as an independent event, again using FLAME as described above. Age and sex were regressed, and results were corrected for multiple comparisons with FSL’s “easythresh” command, a cluster Z threshold >2.3, and a cluster probability threshold of p < 0.01.

### Resting state connectivity analysis

To assess differences in connectivity between CON and CBP participants, we measured modular connectivity of the brain using Matlab scripts from the Brain Connectivity Toolbox [62]. Modular connectivity is a measure of connectivity which essentially clusters regions together based off their direct connections and those of their neighbors. It divides a connectivity matrix into subdivisions (or modules) such that the number of within-module links is maximized, and the number of across-module links is minimized. The resultant modular structure that is extracted from this calculation thus informs which groups of brain regions are most strongly connected to each other overall. As we are focused specifically on the NAc in this study, we use modular connectivity as a tool to narrow down which of all brain regions are most likely functionally related to it.

We first registered functional images to standard MNI space using FSL FLIRT [63], and extracted the mean BOLD time series from 110 anatomical regions of interest (ROI) defined by the FSL Harvard-Oxford cortical and subcortical atlases (excluding the brain stem and anterior/posterior inferior temporal gyrus due to lack of consistent scan coverage across participants). BOLD signals from homologous ROIs were averaged across hemispheres, resulting in 53 total BOLD time series for each subject. BOLD time series were band-pass filtered (0.01 to 0.08 Hz) with a 4th order butterworth filter. Connectivity matrices were then generated for each subject by calculating the pair-wise Pearson correlation between each ROI and performing a Fisher’s z transform, resulting in a 53×53 connectivity matrix. Matrices were then z-scored (to account for overall differences in mean connectivity between subjects). Modular connectivity for weighted matrices was then calculated on each connectivity matrix. As modular connectivity can vary slightly from run to run due to heuristics in the toolbox algorithm, the Louvian modularity of each connectivity matrix was calculated 1000 times, each iteration with an additional Kernighan-Lin fine-tuning to refine the modular structure [64]. The modular structure that appeared most often out of all iterations was chosen to represent the modular connectivity of each participant’s connectivity matrix. Subsequent analyses focused only on the module containing the NAc.

To compare the difference in each region’s membership within the NAc module between groups, we used a nonparametric permutation test similar to ones found in previous literature [65]. The difference between the number of participants who incorporated a given ROI into the NAc module (module 3) was calculated as the actual group difference for that ROI. Then the combined pool of the groups was resampled into two new groups and the mean of these new groups was calculated; this process was repeated for 50,000 iterations in order to create a random null distribution of the difference of the group means. The p-value for the actual group differences for each region was calculated as the percentile in the generated null distribution (p-values <0.05 were considered statistically significant). These values were not corrected for multiple comparisons.

To compare the modular structure of our subjects’ brains to that of another impulsivity study [29], we calculated mutual information (defined by the Brain Connectivity Toolbox), and overlap. Mutual information, ranging from 0 to 1, is a measure that calculates the similarity of the modular divisions across different brains, where a “1” would indicate they are identical. Here, ‘overlap’ is defined as follows: let A = regions in module A, let B = regions in module B, then overlap = [# of regions in A∩B]/[# regions in A U B]. We designate the nomenclature of modules according to the Davis study and follow the labeling of their “intermediate” impulsive subjects. In that study, “module 1” consisted primarily of visual processing regions; “module 2” was mostly somatosensory, motor, and auditory; “module 3” (also labeled “subcortical drive 1”) was the NAc module, but also contained frontal and temporal lobe regions; and “module 4” (also labeled “subcortical drive 2”) consisted mostly of medial temporal lobe structures including the amygdala and hippocampus. For a complete description, see the study cited above.
